# Exploring the retention properties of CaF_2_ nanoparticles as possible additives for dental care application with tapping-mode atomic force microscope in liquid

**DOI:** 10.3762/bjnano.5.4

**Published:** 2014-01-13

**Authors:** Matthias Wasem, Joachim Köser, Sylvia Hess, Enrico Gnecco, Ernst Meyer

**Affiliations:** 1 Department of Physics, University of Basel, Klingelbergstrasse 82, Basel 4056, Switzerland; 2 Institute for Chemistry and Bioanalytics, University of Applied Sciences and Arts Northwestern Switzerland, Muttenz 4132, Switzerland; 3 GABA International AG, Grabetsmattweg, 4106 Therwil, Switzerland; 4 Instituto Madrileño de Estudios Avanzados, IMDEA Nanociencia, 28049 Madrid, Spain

**Keywords:** AM-AFM in liquid, nanodentistry, nanoparticles

## Abstract

Amplitude-modulation atomic force microscopy (AM-AFM) is used to determine the retention properties of CaF_2_ nanoparticles adsorbed on mica and on tooth enamel in liquid. From the phase-lag of the forced cantilever oscillation the local energy dissipation at the detachment point of the nanoparticle was determined. This enabled us to compare different as-synthesized CaF_2_ nanoparticles that vary in shape, size and surface structure. CaF_2_ nanoparticles are candidates for additives in dental care products as they could serve as fluorine-releasing containers preventing caries during a cariogenic acid attack on the teeth. We show that the adherence of the nanoparticles is increased on the enamel substrate compared to mica, independently of the substrate roughness, morphology and size of the particles.

## Introduction

Amplitude-modulation atomic force microscopy (AM-AFM), also known as tapping mode AFM, is a variant of scanning probe microscopy. In this dynamic technique imaging is achieved while a microcantilever is driven at its resonance frequency and the supported probing tip touches the sample surface at the bottom of each oscillation cycle. This imaging mode offers the opportunity to investigate surface structures with gentle force, which for example is required to investigate polymers or biomolecules. Compared to contact mode AFM the destructive lateral forces are virtually eliminated in tapping mode as the probing tip has a much lower contact time while mapping the surface, which results in a much more gentle sensing of the investigated surface [1–2]. AM-AFM has the ability to measure simultaneously the surface morphology and the compositional variations of the mapped surface. These variations are detected by recording the phase-lag of the excitation signal with respect to the vibrating tip, which is known as phase imaging technique. These so-generated phase images are closely related to energy dissipation maps [3–4]. While phase imaging in ambient with high quality cantilever Q-factors is well established [5], a comprehensive model of the energy dissipation process in liquid is still missing since the first studies of AM-AFM measurements in liquid [6–7]. Recent studies have related the phase contrast, when measuring in liquid in which low Q-factors are found, to two origins: the excitation of higher eigenmodes and the energy dissipation on the sample surface [8–9].

In this work we show that for surface associated manipulation of nanoparticles in liquid, the phase-lag in AM-AFM is closely related to the retention properties of the adsorbed nanoparticles, i.e., the particle–substrate contact area and the intrinsic chemical affinity between them. This enabled us to qualitatively compare the adhesion strength of as-synthesized CaF_2_ nanoparticles adsorbed on mica and on tooth enamel in liquid. Manipulation experiments of nanoparticles are routinely done by using the AFM in the contact mode [10–12]. However some studies have been reported, in which a controlled manipulation of nanoparticles in tapping mode AFM was performed. Sitti et al. used a cantilever probe in the dynamic mode to manipulate as-synthesized latex nanoparticles on Si in ambient [13]. Other authors manipulated antimony nanoparticles [14] and gold nanoparticles [15] on graphite also under ambient conditions. Mougin and co-workers moved as-synthesized and functionalized gold nanoparticles on silicon substrates with dynamic AFM [16]. Darwich et al. investigated the retention of colloidal gold nanoparticles depending on particle–substrate affinity and humidity with tapping mode AFM [17]. In all these studies the major difficulty arises to quantify the dynamic processes during manipulation, i.e., the collision between the probing tip and the particle, the friction between the particles and the substrate, the role of water when measuring in ambient (lubrication, capillary effects, etc.), electrostatics between them, etc. The high surface to volume ratio of nanoparticles makes them very interesting for application in science, technology and medical applications including dentistry [18]. In this context calcium fluoride is of high interest in saliva chemistry and in the context of reducing acid dissolution of teeth [19]. The outermost layer of the teeth, also called enamel, has the purpose to protect the inner sensitive part of the teeth from physical or chemical attacks. It consists of tightly packed hydroxyapatite crystals and has a thickness of up to 2.5 mm. The solubility of enamel depends highly on the pH value [20]. The consumption of acidic beverages directly lowers the pH value in the vicinity of teeth while bacteria in the dental plaque metabolize sugars and lower the pH value on the tooth surface. If the pH drops below a certain threshold value the tooth enamel starts to dissolve. This demineralization process of the enamel is better known as enamel erosion and caries disease. The use of CaF_2_ nanoparticles as a source of fluoride in order to prevent caries was already discussed in early studies [19,21–22]. Little research to explore tooth enamel has been done with AFM. Studies investigated the erosion of enamel with AFM based nanoindentation and related the demineralization and remineralization processes to softening of the enamel [23–24]. Another study recorded force–distance curves with AFM tips on etched superficial enamel substrate to examine the softening of enamel [25]. The formation of fluoride-containing nanostructures on tooth enamel upon exposure to a fluorated solution has been observed with AFM in liquid [26]. To the best of our knowledge no manipulation experiment of particles adsorbed on human tooth enamel has been performed so far. The anticaries activity of calcium fluoride nanoparticles is mainly determined by two factors, the solubility of the nanocomposites at a certain pH value and their adhesion strength to the tooth enamel upon application. The solubility of CaF_2_ has already been investigated by titration methods with respect to the pH-dependent fluoride release [27]. In this study we focus on exploring the adhesion strength of calcium fluoride nanoparticles adsorbed on mica and on tooth enamel in liquid with AM-AFM.

## Theory

**Figure 1:**
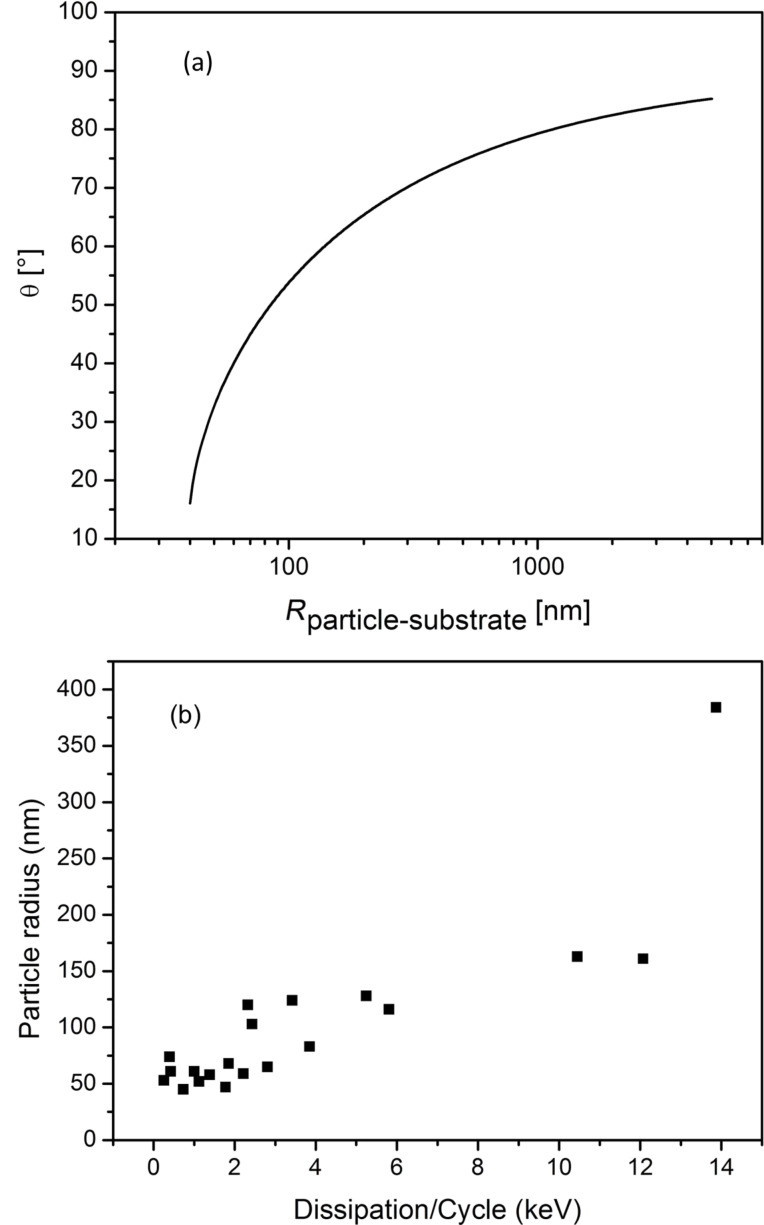
(a) Theoretically predicted trajectory angles of nanoparticles manipulated on an arbitrary surface as a function of the particle–substrate contact radius with *b* = 39 nm. (b) Calculated particle radius and energy dissipation obtained by using Equation 2 with an experimentally determined phase-lag and trajectory angle of the manipulated particles adsorbed on mica. The amplitude was *A* = 23 nm, the Q-factor = 7 and *A*_0_ = 1.2*A*.

As already described above, manipulation experiments in the tapping-mode are difficult to quantify as dynamic and friction processes are involved at the same time. To connect the power dissipation to the particle–substrate interplay we used the work of two earlier studies. To quantify the particles–substrate contact we use the relation derived from Rao et al. [28]. They showed that when performing AM-AFM manipulation experiments with nanoparticles in the raster scan path, the particles are deflected in a direction defined by the geometries of the probing tip, the particles contact area with the substrate and the spacing between consecutive scan lines. If the radius of the nanoparticles is very big compared to the tip radius, and the spacing between the scan lines, *b*, remains constant, the trajectory angle of the manipulated particle is mainly a function of the intrinsic particle–substrate contact radius *R*. With exception for the first scan line, the displacement angle of a particle *θ* with respect to the fast scan axis is given by:

[1]
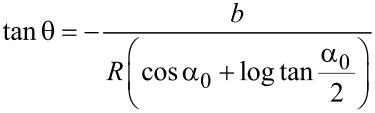


where α_0_ = arcsin [1−(*b*/*R*)]. The theoretical predicted deflection angle depending only on the particle-substrate contact radius derived from Equation 1 is illustrated in Figure 1a.

By reading out the trajectory angle of the deflected particles we get the particle–substrate contact size distribution. For the case of particles with plane facing adsorbed on smooth and atomically flat substrates, such as mica, the trajectory angle distributions can be approximately regarded as the size distribution of the synthesized particles. In order to calculate the power dissipation from the phase-lag of the cantilever relative to the excitation, we used the method of Cleveland et al. [3]. In this method, in the dynamic steady-state equilibrium, the average rate at which energy is fed into the cantilever must equal the average rate at which energy is dissipated by the cantilever and the tip. With this restriction one can separate the total dissipated power into two terms, 
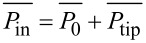
. The first term of the dissipated power, 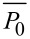, can be thought as the average power dissipated by the body of the cantilever (i.e., air damping or in our case damping of the cantilever motion in the liquid) and can be modeled by simple viscous damping. The second part, 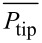, corresponds to the power dissipated by tip–sample interactions. If the cantilever has a normal spring constant *k* and is driven sinusoidally with the amplitude *A*_0_ and drive frequency ω_0_, we can calculate the average power dissipated by tip–sample interactions as

[2]
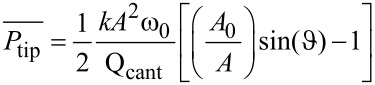


where *A* is the damped amplitude at the given set point, Q_cant_ the quality factor of the cantilever and 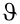 the phase angle. According to this equation the power lost by tip–sample interaction is proportional to the sine of the phase-lag. It is important to note that Equation 2 allows to calculate the total energy lost by tip–sample interactions but does not reveal how it is lost.

## Experimental

### Synthesis of CaF_2_ nanoparticles

**Figure 2:**
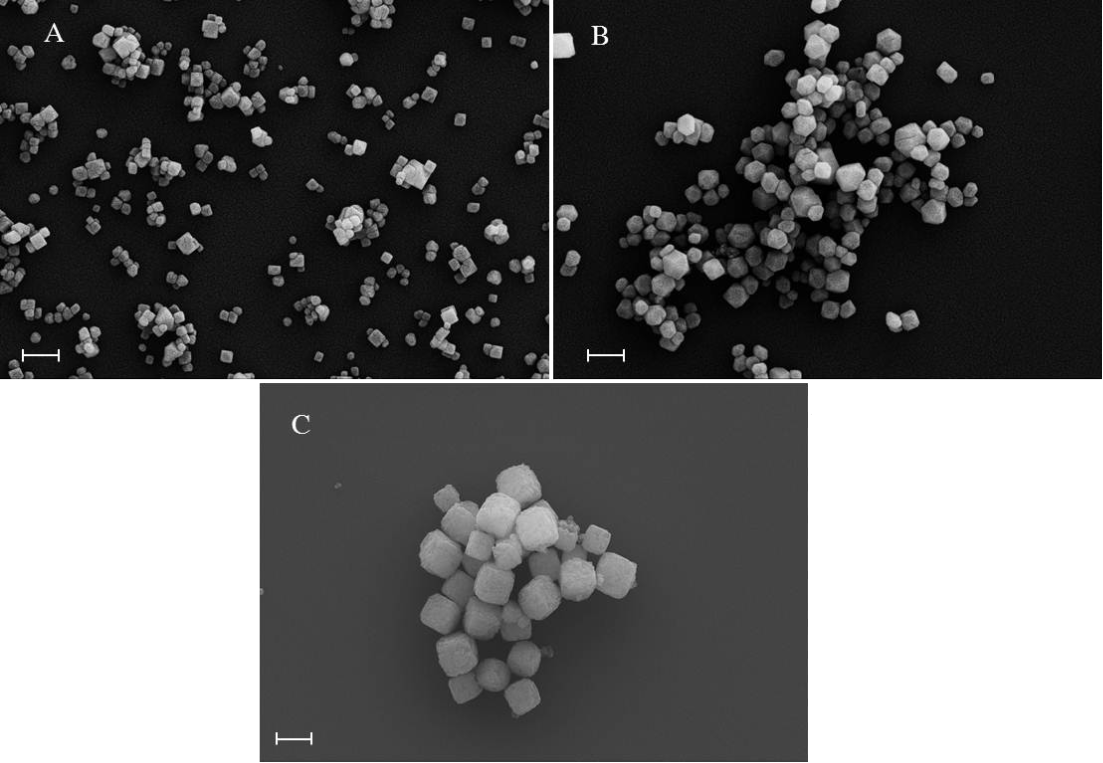
SEM images of the three morphologies of nanoparticles explored in this work. A certain size distribution of the particles was achieved with the synthesis method described in the text. The diameters vary from 50–100 nm for (A), 100–150 nm for (B) and 200–250 nm for (C). The shape varied from cubic (A) to polyhedral (B) and oblate cubic (C). The scale bar for all images is 200 nm.

The CaF_2_ nanoparticles were synthesized through a procedure called the precipitation method [29]. Particles with defined morphology were prepared at room temperature by preparing a 1:1 volumetric mixture of unbuffered aqueous CaCl_2_ and NaF salt solutions with specific concentrations. The formation process of the particles was very fast as the solution became rapidly opaque. Generally no differences were observed if particles were assembled overnight or for several days. The formed nanoparticles were purified by centrifugation and washed several times with a saturated solution of calcium fluoride to remove excess salt ions. Subsequently the nanoparticles were vacuum dried, resulting in a white powder, which was stored in a dry and dark environment until use. Scanning electron microscopy (SEM) was used to determine the size and shape of the particles. The three types of CaF_2_ nanoparticles examined in this work have been prepared by mixing a) 50 mM NaF and 250 mM CaCl_2_ (Figure 2a), b) 50 mM NaF and 50 mM CaCl_2_ (Figure 2b) and c) 75 mM NaF and 1 mM CaCl_2_ (Figure 2c).

### Sample preparation and manipulation experiments with AFM

**Figure 3:**
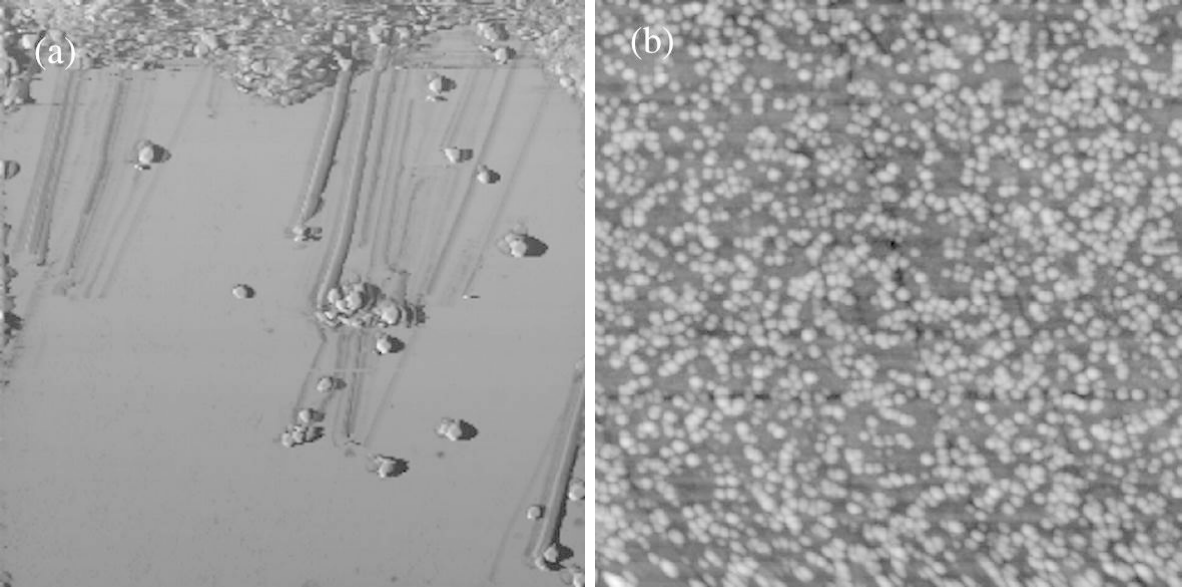
(a) Phase image of nanoparticles (B) adsorbed and manipulated on mica substrate. (b) Topography image of nanoparticles (A) adsorbed on enamel substrate. Scan size is for both images 10 μm.

The adsorption of CaF_2_ nanoparticles on the sample surface was performed as follows. An aliquot of the dried nanoparticles was mixed with 200 μL saturated calcium fluoride solution and put in an ultrasonic bath for 20 min to break any aggregated particles. Subsequently, 2 μL were dropped onto the substrate and dried. All measurements were performed in a saturated solution of calcium fluoride with pH 6, to inhibit any change of the adsorbed nanoparticles. The mica substrate was freshly cleaved prior to use. Human wisdom teeth embedded in resin were generously donated from GABA International (Therwil, Switzerland). The enamel was polished with 3 μm and 1 μm diamond paste grain size under constant water cooling. The cleaning procedure of the polished tooth enamel was done as described elsewhere [30]. All specimens were stored in a dust-free box and not further processed. A topography image of nanoparticles (A) adsorbed on enamel is illustrated in Figure 3b.

All imaging and manipulation experiments were performed by using a commercially available AFM (Flex AFM from Nanosurf AG, Switzerland). Rectangular silicon cantilevers with typical resonance frequencies in air and liquid of 160 kHz and 70 kHz, respectively, and spring constants of 45 N·m^−1^ and 7 N·m^−1^, respectively, have been used (PointProbe PPP-NCLPt from Nanosensors AG, Switzerland). A typical phase image obtained after a manipulation experiment is shown in Figure 3a. To compare the manipulation experiments done on mica and on tooth enamel we conducted measurements with comparable amplitudes and set points.

## Results and Discussion

**Figure 4:**
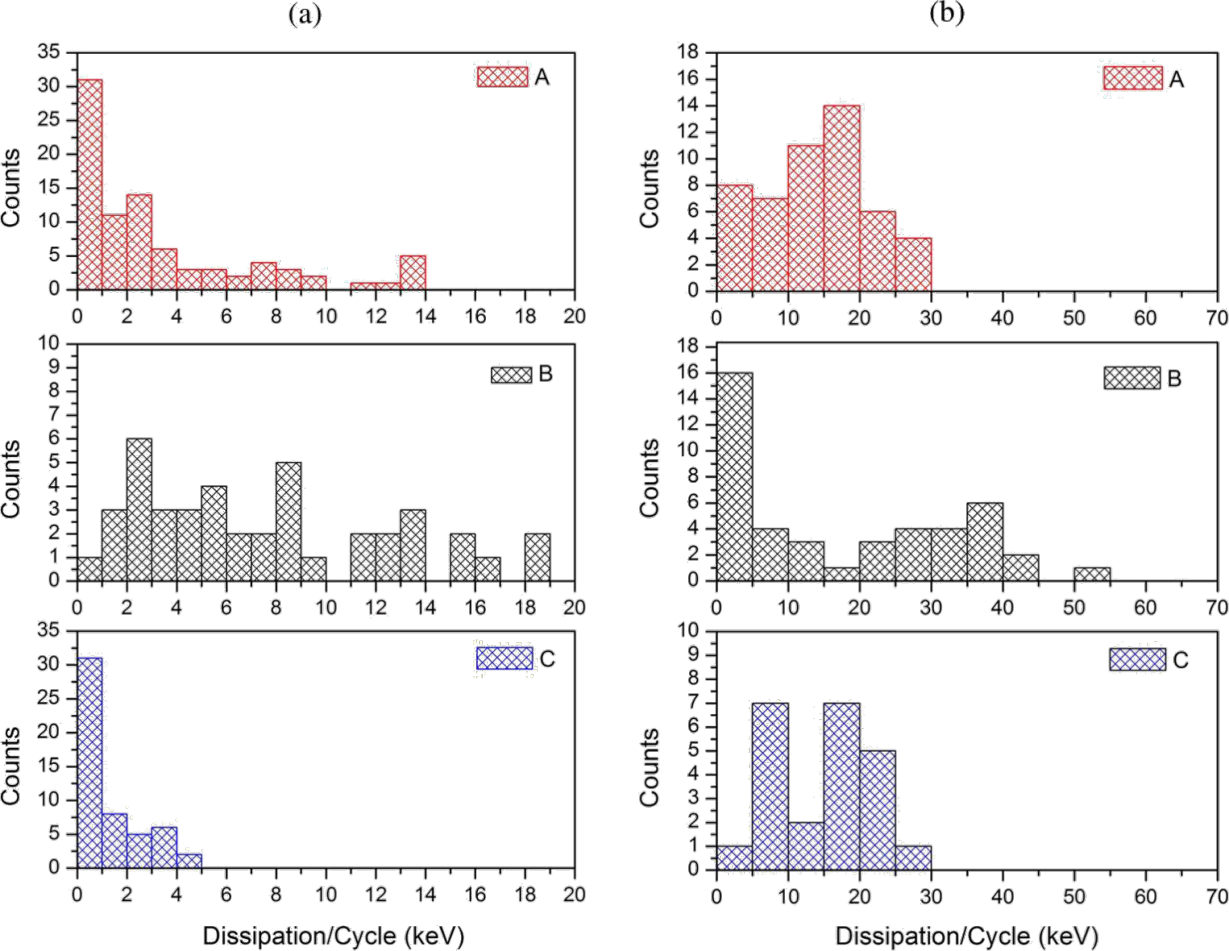
Calculated energy dissipation histograms obtained for the nanoparticles A, B and C from Figure 3 on mica (a) and tooth enamel (b). The dissipated power was calculated per oscillation cycle of the cantilever. Amplitude and set point were comparable for all experiments.

To address the calculated power dissipation to the retention of the nanoparticles on a given surface, i.e., the particle–substrate contact area and the chemical interplay between them, we recorded the phase-lag at the point the particles were displaced and their trajectory angle relative to the fast scan direction. In Figure 1b a plot is illustrated for the nanoparticles (A) obtained from one single manipulation experiment. The result was reproducible for all nanoparticles. As discussed above the trajectory angle of manipulated particles is closely related to the nanoparticle–substrate contact area. The determined particle radius from the trajectory angle for the manipulated specimen (A) confirms the radius distribution observed from the SEM images in Figure 2 of *r* = 50–100 nm. The size distribution of CaF_2_ particles synthesized with the precipitation method described above depends on following parameters: the degree of supersaturation of the solution, the spatial concentration distribution and the growth time of the crystals [31]. Not precisely controlling these factors in our synthesis procedure resulted in a certain size distribution of the synthesized particles. As the facets of particles (A) are smooth and plane the particles radius equals the contact radius with the substrate. It also shows that at higher contact radii, more energy is needed to dislocate the particle. The phase-lag correlates with the power that is needed to move the particles. This correlation between energy dissipation and deflection angle was observed for all three kinds of nanoparticles investigated. Manipulation experiments in liquid have the advantage, compared to measurements in ambient, that the retention of adsorbed particles in not dominated by the wetting layer (hydrodynamic drag of water layer), but the intrinsic particle–sample interaction energies. The frequency distribution of the calculated power dissipated for each of the particles enables us to examine the chemical affinity between the calcium fluoride nanoparticles adsorbed on mica and on polished tooth enamel. The energy dissipation histograms obtained for the three nanoparticles are illustrated in Figure 4.

The histograms in Figure 4a show the distribution of the calculated power dissipated for CaF_2_ nanoparticles adsorbed on mica. The lowest energy dissipation was found for the specimen (C) with a power dissipation per cycle of the cantilever of around 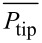 = 1 keV/cycle. These particles were the biggest in size that we examined with an approximate diameter of *d* = 200–250 nm. The fact that they show the least energy that was needed to manipulate them, is related to the rough and spherical surface structure of these particles. Compared to the smaller particles (A) and (B), which have plane and smooth faceting, the high surface roughness leads to a smaller contact area on the substrate. The fact that the lowest energy dissipation was observed for specimen (C) is consistent with the number of asperity contacts in the interface of the particle–substrate system. Comparing particles (A) and (B) we find comparable dissipated powers needed to displace the particles. The broad distribution of the power dissipation for the specimen (A) and especially (B) can be explained by the fact that more conglomerated particles were moved instead of isolated single ones. Nevertheless, the smaller particles with a diameter ranging from 50–150 nm and with smooth and plane surfaces show a higher retention as big particles with rough and spherical facing. The histograms in Figure 4b show the distribution of the calculated power dissipated for nanoparticles adsorbed on polished tooth enamel substrates. The relatively wide distribution of the calculated dissipated energies for all three particles may arise from the inhomogeneous scratch profiles for each of the polished tooth enamel substrates used. Remarkably, the energy dissipation for each of the particles is found to be much higher on enamel than on mica. For particles (C) the power dissipation is found to be up to 10-times higher on tooth enamel. The specimens (A) and (B), which are similar in size and surface structure show an increase in the dissipated power of around 5 to 10 times on tooth enamel compared to the adsorption on mica. The interaction between particles and a substrate is known to depend on the size of the contact area, i.e., the nanoparticle- and substrate surface roughness and geometry. Compared to mica, which is atomically flat, the tooth enamel, which was mechanically polished before use, had a mean square roughness (RMS) ranging between 3.4–4.0 nm. The higher substrate roughness of enamel would lower the particle–substrate contact area. This has been experimentally verified in earlier studies by measuring the pull-off force of nanoparticles attached to a cantilever tip on surfaces of different roughness [32–33]. Recent studies have simulated that the mobility of nanoparticles is enhanced on rough surfaces, compared to smooth ones, if the asperities are much smaller than the particles [34–35]. This phenomenon was explained in terms of less contacting asperities in the substrate–particles interface and hence less adhesion force acting between them. Our experiments show the exact opposite behavior, at a higher substrate roughness we observe a higher retention of the nanoparticles. We explain this in terms of the higher chemical affinity of the calcium fluoride nanoparticles to the tooth enamel substrate. Earlier studies that examined the enamel surface chemistry at certain pH values that were varied between 2 and 10, have shown that the enamel surface is covered with distinct ionic species depending on the pH value, which come from the enamel and the ionic species present in the liquid [36–38]. In our case, we speculate that the surface polarity of the enamel strongly influenced the retention of the adsorbed CaF_2_ nanoparticles. The matching of the polarity of the substrate and the nanoparticle resulted in an increased adhesion strength between the particle and the substrate despite the decreased number of contacting asperities.

## Conclusion

To conclude, we compared the retention properties of as-synthesized CaF_2_ nanoparticles adsorbed on mica and on polished tooth enamel in liquid. From the phase-lag of the cantilever with respect to the excitation signal we calculated the power dissipation at the point the nanoparticles were mobilized. By comparing the frequency distribution of the obtained dissipated power, we showed that an up to ten times higher retention was observed for particles adsorbed on tooth enamel compared to mica. Although the enamel had an increased surface roughness compared to mica as a result of the mechanical polishing, which resulted in a decreased contact area of the particle with the substrate, more power was needed to dislocate the particles. We relate this to the strong chemical interaction of the CaF_2_ nanoparticles with the tooth enamel. Further, we have shown that particles with an ordered, smooth and plane surface structure show a higher retention than rough and spherical ones. Thus, the nano-morphology of particles has a strong influence on the mobility. The evidence that the interplay of calcium fluoride nanoparticles with the tooth enamel is so strong, makes calcium fluoride nanoparticles a promising candidate to be used in dental care products preventing teeth demineralization. Regarding the solubility of the CaF_2_ nanoparticles, further experiments are needed to examine how the retention varies with respect to the pH and also solubility test are required to explore their acid-dependent release of fluorine over time. We showed that AM-AFM is a powerful tool to compare detachment and interaction properties of adsorbates in liquid.

## References

[1] Tamayo J, García R (1996). Langmuir.

[2] Magonov S N, Elings V, Whangbo M-H (1997). Surf Sci.

[3] Cleveland J P, Anczykowski B, Schmid A E, Elings V B (1998). Appl Phys Lett.

[4] Anczykowski B, Gotsmann B, Fuchs H, Cleveland J P, Elings V B (1999). Appl Surf Sci.

[5] Tamayo J, García R (1998). Appl Phys Lett.

[6] Hansma P K, Cleveland J P, Radmacher M, Walters D A, Hillner P E, Bezanilla M, Fritz M, Vie D, Hansma H G, Prater C B (1994). Appl Phys Lett.

[7] Putman C A J, Van der Werf K O, De Grooth B G, Van Hulst N F, Greve J (1994). Appl Phys Lett.

[8] Melcher J, Carrasco C, Xu X, Carrascosa J L, Gómez-Herrero J, de Pablo P J, Raman A (2009). Proc Natl Acad Sci U S A.

[9] Payam A F, Ramos J R, Garcia R (2012). ACS Nano.

[10] Dietzel D, Mönninghoff T, Jansen L, Fuchs H, Ritter C, Schwarz U D, Schirmeisen A (2007). J Appl Phys.

[11] Palacio M, Bhushan B (2008). Nanotechnology.

[12] Dietzel D, Feldmann M, Herding C, Schwarz U D, Schirmeisen A (2010). Tribol Lett.

[13] Sitti M, Hashimoto H (2000). IEEE/ASME Trans Mechatronics.

[14] Ritter C, Heyde M, Stegemann B, Rademann K, Schwarz U D (2005). Phys Rev B.

[15] Paolicelli G, Rovatti M, Vanossi A, Valeri S (2009). Appl Phys Lett.

[16] Mougin K, Gnecco E, Rao A, Cuberes M T, Jayaraman S, McFarland E W, Haidara H, Meyer E (2008). Langmuir.

[17] Darwich S, Mougin K, Rao A, Gnecco E, Jayaraman S, Haidara H (2011). Beilstein J Nanotechnol.

[18] Uskoković V, Bertassoni L E (2010). Materials.

[19] Xu H H K, Moreau J L, Sun L, Chow L C (2010). J Dent Res.

[20] Larsen M J, Jensen S J (1994). Arch Oral Biol.

[21] Xu H H K, Weir M D, Sun L, Moreau J L, Takagi S, Chow L C, Antonucci J M (2010). J Dent Res.

[22] Azami M, Jalilifiroozinezhad S, Mozafari M, Rabiee M (2011). Ceram Int.

[23] Lippert F, Parker D M, Jandt K D (2004). J Colloid Interface Sci.

[24] Cross S E, Kreth J, Wali R P, Sullivan R, Shi W, Gimzewski J K (2009). Dent Mater.

[25] Pelin I M, Piednoir A, Machon D, Farge P, Pirat C, Ramos S M M (2012). J Colloid Interface Sci.

[26] Petzold M (2001). Caries Res.

[27] Pan H-B, Darvell B W (2007). Arch Oral Biol.

[28] Rao A, Gnecco E, Marchetto D, Mougin K, Schönenberger M, Valeri S, Meyer E (2009). Nanotechnology.

[29] Dirksen J A, Ring T A (1991). Chem Eng Sci.

[30] Lussi A, Jaeggi T, Gerber C, Megert B (2004). Caries Res.

[31] Chen J-F, Wang Y-H, Guo F, Wang X-M, Zheng C (2000). Ind Eng Chem Res.

[32] Beach E R, Tormoen G W, Drelich J, Han R (2002). J Colloid Interface Sci.

[33] Götzinger M, Peukert W (2004). Langmuir.

[34] Korayem M H, Zakeri M (2011). Appl Surf Sci.

[35] Klapetek P, Valtr M, Nečas D, Salyk O, Dzik P (2011). Nanoscale Res Lett.

[36] Robinson C, Connell S, Brookes S J, Kirkham J, Shore R C, Smith D A M (2005). Arch Oral Biol.

[37] Robinson C, Yamamoto K, Connell S D, Kirkham J, Nakagaki H, Smith A D (2006). Eur J Oral Sci.

[38] Harding I S, Rashid N, Hing K A (2005). Biomaterials.

